# Editing the *CsDMR6* gene in citrus results in resistance to the bacterial disease citrus canker

**DOI:** 10.1093/hr/uhac082

**Published:** 2022-04-11

**Authors:** Saroj Parajuli, Heqiang Huo, Fred G Gmitter, Yongping Duan, Feng Luo, Zhanao Deng

**Affiliations:** 1 Gulf Coast Research and Education Center, University of Florida, IFAS, 14625 County Road 672, Wimauma, FL 33598, USA; 2 Mid-Florida Research and Education Center, University of Florida, IFAS, 2725 S. Binion Road, Apopka, FL 32703, USA; 3 Citrus Research and Education Center, University of Florida, IFAS, 700 Experiment Station Road, Lake Alfred, FL 33850, USA; 4 USDA-ARS, U.S. Horticultural Research Laboratory, 2001 South Rock Road, Fort Pierce, FL 34945, USA; 5 Clemson University, School of Computing, 100 McAdams Hall, Clemson, SC 29643, USA

Dear Editor,

Citrus (*Citrus* L.) is one of the most important fruit crops in the world. Sweet oranges (*C*. × *sinensis*), grapefruit (*C*. × *paradisi*), pummelos (*Citrus maxima*), lemons (*C*. × *limon*), limes (*Citrus aurantifolia*), mandarins (*Citrus reticulata*), and tangerines (*Citrus reticulata*) are the major cultivated types of *Citrus*. Production worldwide faces challenges from devastating bacterial diseases, including citrus canker, citrus greening (Huanglongbing, HLB), and citrus variegated chlorosis [2]. Citrus canker is caused by *Xanthomonas citri* ssp. *citri* (Xcc); most citrus species and cultivars are susceptible to citrus canker. Improving resistance to citrus canker has been an important citrus breeding objective. Recent studies have shown that disabling disease susceptibility genes including the *DOWNY MILDEW RESISTANCE* 6 (*DMR6*) gene can be a promising approach to engineering resistance to diseases [1, 3, 5, 6].

*DMR6* is a repressor of plant immunity [9] that negatively regulates the expression of plant defense genes. The expression of *DMR6* is required for plant susceptibility to pathogen *Hyaloperonospora*, *Pseudomonas syringae* pv. *Tomato* DC3000 [10], and *Phytophthora capsici* [7]. Mutagenesis of tomato *DMR6* resulted in broad-spectrum resistance to *Pseudomonas syringae*, *Phytophthoracapsica*, *Xanthomonas gardneri*, and *perforans* [1]. *DMR6* may be involved in citrus susceptibility to HLB [4, 8]. Here, we demonstrate that editing *CsDMR6* in two *Citrus* cultivars, “Duncan” grapefruit and Carrizo citrange (*C.* × *sinensis* × *Poncirus trifoliata*), results in strong resistance to citrus canker.

Two guide RNAs [dmr6-gRNA1 (***CCT***CGGGAATCCGGTACACAAAC), and dmr6-gRNA2 (AGTGGAAAGAGTCTTAGAAG***TGG***)] were designed to target *CsDRM6*. Both gRNAs have 100% identity with *DMR6* in sweet orange, mandarins, limes, pummelo, and trifoliate orange (*P. trifoliata*). A plasmid vector (Figure 1A) was constructed to express the gRNAs and the HypaCas9, GFP and NPTII genes, introduced into *Agrobacterium tumefaciens* EHA101, and then transferred into citrus through *Agrobacterium-*mediated transformation (five and nine transformation experiments for “Duncan” and Carrizo, respectively). Co-cultivation of 1900 “Duncan” and 5320 Carrizo epicotyl segments with *Agrobacterium* followed by kanamycin and green fluorescence protein (GFP) selection resulted in nine and 57 GFP-positive shoots, respectively. Based on the GFP expression, the transformation efficiency with “Duncan” and Carrizo was 0.47% and 1.07%, respectively, which were lower than those previously reported. One possible cause of the lower transformation efficiencies might be the much larger size of the T-DNA region in this gene-editing vector. Micro-grafting of GFP-positive shoots resulted in four “Duncan” and 16 Carrizo GFP-positive complete plants in soil. Out of these complete plants, two “Duncan” (DD9 and DD19) and four Carrizo lines (D4, D7, D10, and D12) were analyzed for induced mutations and resistance to Xcc.

**Figure 1:**
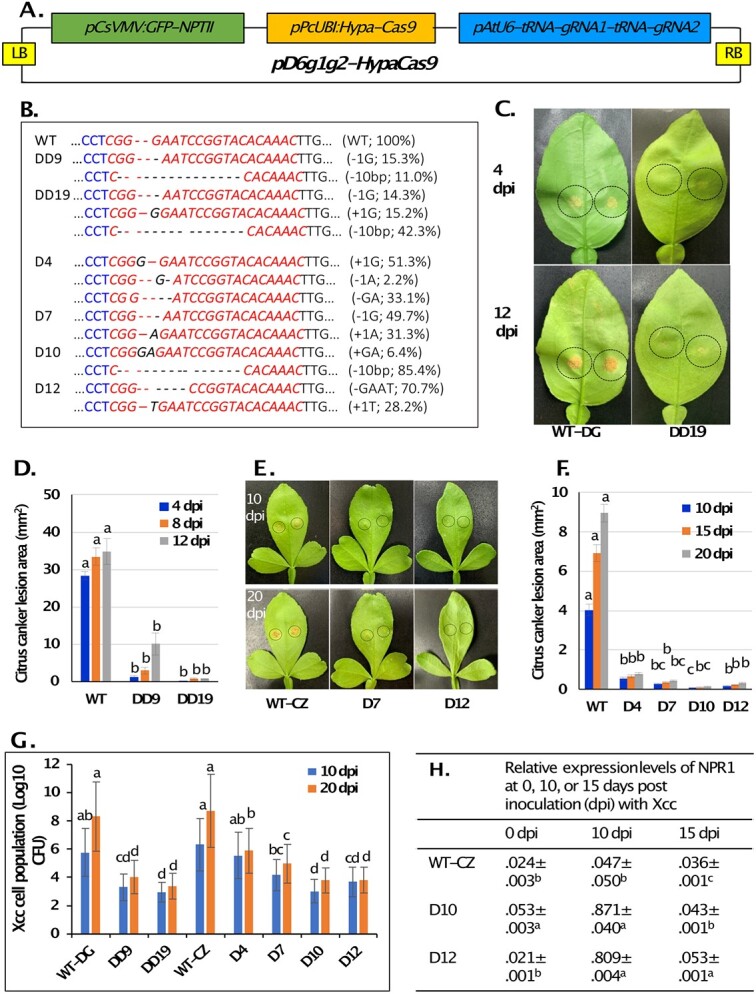
Citrus *dmr6* mutants show resistance to citrus canker caused by *Xanthomonas citri* ssp. *citri* (Xcc). A. Schematic map of plasmid vector for editing *CsDMR6*. B. DNA sequences of wildtype (WT) and *dmr6* mutants at the first gRNA-targeted region, mutations, and mutation frequencies (in parenthesis) in “Duncan” and Carrizo. C and D. Citrus canker lesions (C) (within the dotted circles) and lesion size (mm^2^) (D) on leaves of wildtype “Duncan” grapefruit (WT-DG) and mutant DD19 at 4, 8 (in D only), and 12 days post inoculation (dpi). E and F. Citrus canker lesions (E) (within the dotted circles) and lesion size (mm^2^) (F) on leaves of wildtype Carrizo (WT-CZ) and mutant D7 and D12 at 10, 15 (in F only) and 20 dpi. G. Xcc cell populations (Log10 of colony-forming units per cm^2^) in leaves of wildtype “Duncan” (WT-DG) and Carrizo (WT-CZ) and mutants DD9, DD19, D4, D7, D10, and D12) at 10 and 20 dpi. H. Relative expression of *NPR1* in leaves of wildtype Carrizo and mutants D10 and D12 prior to and 10 and 15 dpi. The glyceraldehyde 3-phosphate dehydrogenase (*GAPDH*) gene was used as a reference gene to calculate the relative gene expression level of *NPR1* using the 2^-ΔΔ(Ct)^ method.

Three rounds of deep amplicon sequencing analysis of multiple branches of each mutant over 18 months revealed similar types and frequencies of mutations in each mutant line but multiple types of mutations and mutation frequencies in different mutant lines (Figure 1B). DD9 contained two primary types of mutations and a mutation frequency of 38.5%. DD19 contained three primary types of mutations and showed a mutation frequency of 74.2%. The primary mutations in D4 were one-base insertion and two-base deletion, and this line had a mutation frequency of 89.0%. The primary mutations in D7 were one-base deletion and one-base insertion, and the mutation frequency was 84.6%. The primary mutation in D10 was 10-base deletion, and this mutant had a mutation frequency of 91.8%. The primary mutations in D12 were four-base deletion and seven-base deletion, and D12 showed a mutation frequency of 100%.

Significant differences were observed in the two gRNA-targeted regions in mutation types and frequencies. Nine types of mutations were induced in the six mutants in the dmr6-gRNA1-targeted region, including the deletion of one, two, four, or 10-bases, or the insertion of one or two bases of different nucleotides (Figure 1B). These deletions and insertions resulted in frameshifting of the coding region in *CsDMR6*. The mutation frequency in this region in five out of six mutants ranged from 71.8% (DD19) to 98.9% (D12). Fewer types of mutations (three) and much lower frequencies (2.5% to 12.2%) of mutation were induced in the dmr6-gRNA2-targeted region in most of the mutants.

The abaxial side of immature leaves of these mutants and wildtypes were inoculated with cell suspensions [1 x 10^8^ colony-forming units (CFU)/mL] of Xcc strain 2004–0059, the main strain in Florida, and then citrus canker lesions (mm^2^ per inoculation site) and Xcc bacterial cell populations were measured. Compared to wildtype “Duncan” grapefruit, mutant DD9 and DD19 showed 71.2% and 99.8% reduction in canker lesion 4 to 12 days post inoculation (dpi) (Figure 1C, 1D). The Xcc cell counts in DD9 and DD19 leaves were reduced by >99.7% (2.45 to 4.95 Log10 units) at 10 or 20 dpi (Figure 1G). Compared to wildtype Carrizo, the citrus canker lesion on leaves of D4, D7, D10, and D12 was reduced by 86.5% to 98.7% 10 to 20 dpi (Figure 1E, 1F). The Xcc cell population in the leaves of D4, D7, D10, and D12 were reduced by 99.8% or greater (2.81 to 4.92 Log10 units) at 20 dpi (Figure 1G).

The *non-expressor of pathogenesis-related genes 1* (*NPR1*) acts as the master key to the plant defense signaling network and is essential for establishing systemic acquired resistance. Before *Xcc* inoculation (0 dpi), 15 and 20 dpi, the expression levels of *NPR1* in wildtype Carrizo and mutants D10 and D12 were low, ranging from 0.021 to 0.053. However, by 10 dpi, *NPR1* expression increased by 16–39 fold in both mutants whereas it increased by ~2 fold in wildtype (Figure 1H).

In summary, disruptive (frameshift) mutagenesis of *CsDMR6* resulted in strong resistance to citrus canker, an important bacterial pathogen to the global citrus industry. HypaCas9 mediated high frequencies of mutations in citrus. The guide RNAs reported here can target *DMR6* in multiple important citrus species and cultivars. Functional knocking down of *CsDMR6* increased *NPR1* expression in citrus, thus editing *CsDMR6* may improve citrus resistance to other pathogens.

## Acknowledgements

Financial support from USDA/NIFA/SCRI/CDRE (2017-70016-26051). Dr. Jeff Jones provided Xcc strain2004-00059 and advice on assessing resistance to citrus canker; Mr. Joseph Alexander provided grapefruit seeds.

## Author contributions

SP constructed plasmids, generated mutants, and determined citrus canker resistance and gene expression; HH assisted plasmid construction; ZD, FGG, YD, and FL conceived the project; ZD supervised the project and wrote the manuscript. All authors reviewed, revised, and approved the manuscript.

## Data availability

All data are available in the main text.

## Conflict of Interest Statement

The authors declare no conflict of interests, except that ZD and SP submitted a patent application to the USPTO.
